# Complete genome sequence of *Treponema pallidum* strain DAL-1

**DOI:** 10.4056/sigs.2615838

**Published:** 2012-09-24

**Authors:** Marie Zobaníková, Pavol Mikolka, Darina Čejková, Petra Pospíšilová, Lei Chen, Michal Strouhal, Xiang Qin, George M. Weinstock, David Šmajs

**Affiliations:** 1 Department of Biology, Faculty of Medicine, Masaryk University, Brno, Czech Republic; 2 Human Genome Sequencing Center, Baylor College of Medicine, Houston, Texas, USA; 3 The Genome Institute, Department of Genetics, Washington University School of Medicine, St. Louis, Missouri, USA

**Keywords:** *Spirochaetaceae*, *Treponema pallidum*, syphilis

## Abstract

*Treponema pallidum* strain DAL-1 is a human uncultivable pathogen causing the sexually transmitted disease syphilis. Strain DAL-1 was isolated from the amniotic fluid of a pregnant woman in the secondary stage of syphilis. Here we describe the 1,139,971 bp long genome of *T. pallidum* strain DAL-1 which was sequenced using two independent sequencing methods (454 pyrosequencing and Illumina). In rabbits, strain DAL-1 replicated better than the *T. pallidum* strain Nichols. The comparison of the complete DAL-1 genome sequence with the Nichols sequence revealed a list of genetic differences that are potentially responsible for the increased rabbit virulence of the DAL-1 strain.

## Introduction

*Treponema pallidum* is an uncultivable human pathogen causing the sexually transmitted disease, syphilis. Until now, three syphilis causing strains of *T. pallidum* have been completely sequenced including strain Nichols [1], SS14 [2], and Chicago [3]. In addition, a number of related treponemes causing yaws including strains Samoa D, CDC-2, Gauthier [4] and *T. paraluiscuniculi* strain Cuniculi A [5] have been sequenced. The data indicates that pathogenic treponemes are extremely closely related and small genetic changes can result in profound changes in pathogenesis and host range [6]. The accumulation of genomic data provides new insights into the pathogenesis of treponemal diseases and into the evolution of pathogenic treponemes and brings new opportunities for molecular diagnostics of syphilis [6]. *T. pallidum* strain DAL-1 was isolated using intratesticular injection of rabbits with amniotic fluid taken from a 21-year-old African American woman (at 35 weeks of gestation) in the secondary stage of syphilis [7]. In rabbits, the DAL-1 strain replicated better than the Nichols strain [1,7]. Therefore, the genome sequencing of the DAL-1 strain and its comparison with the Nichols sequence should reveal a list of genetic differences that are potentially responsible for increased rabbit virulence of the DAL-1 strain.

## Classification and features

**Figure 1:**
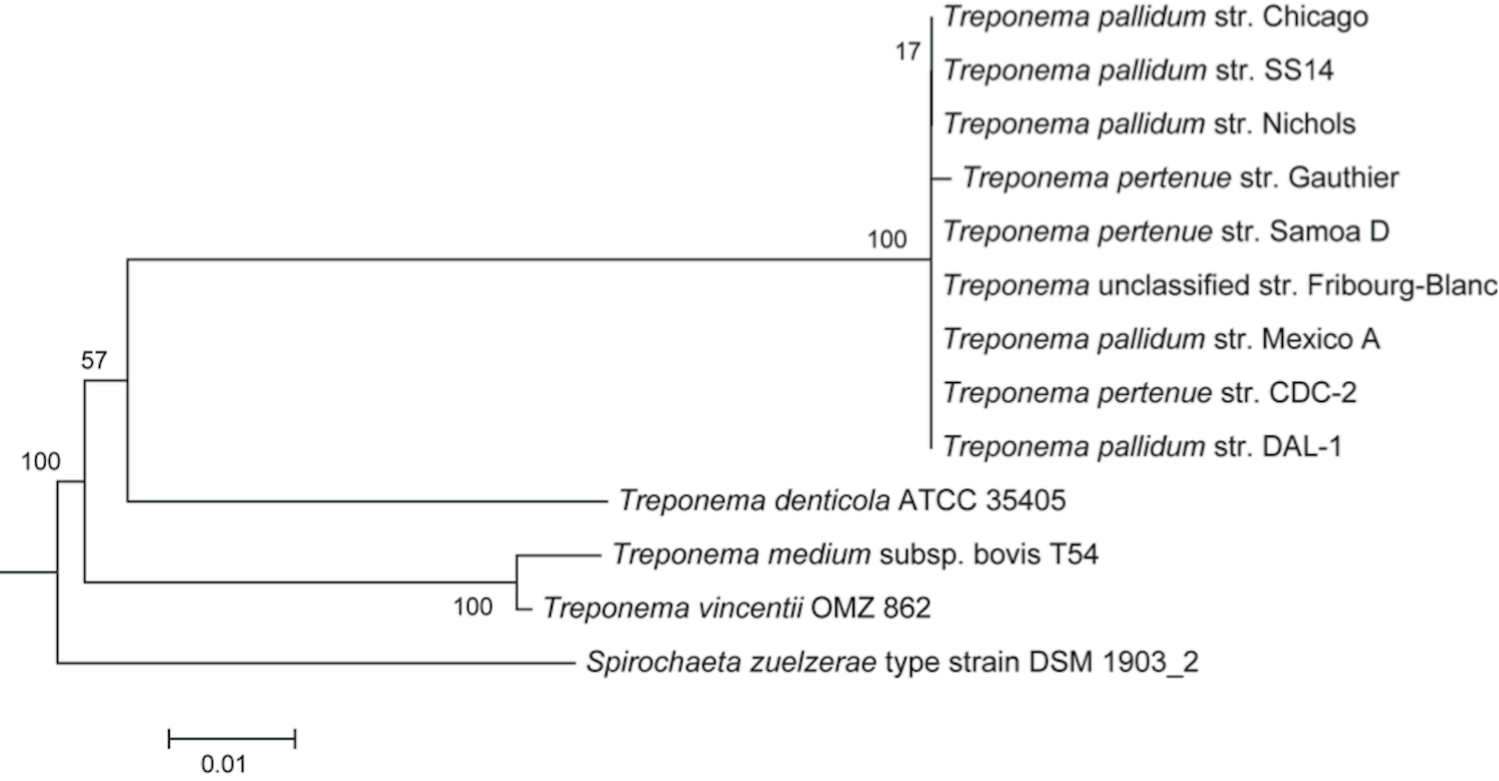
Phylogenetic tree based on 16S rRNA of *T. pallidum* DAL-1 and some strains of *Treponema* species. The bar scale represents the number of nucleotide substitutions per 1 nt site. The tree was generated using tree-builder, which is available from the Ribosomal Database project [28], using the Weighbor (weighted neighbor-joining) algorithm [29] and the Jukes-Cantor distance correction [30]. A *Spirochaeta zuelzerae* type strain was used as the out-group.

*Treponema pallidum*, previously known as *Spirochaeta pallida* [8], is an etiologic agent of syphilis. Based on DNA hybridization studies [9], *Treponema pallidum* and yaws [10] causing *Treponema pertenue* were found to be genetically indistinguishable. The rabbit pathogen, *Treponema paraluiscuniculi*, is not pathogenic to humans and the sequence identity is greater than 98% on a genome wide scale [5]. The genus *Treponema* belongs to the family *Spirochaetaceae* (see Table 1). Genetic relatedness of *T. pallidum* strain DAL-1 to other treponemes and spirochetes is shown in Figure 1.

**Table 1:** Classification and the general features of *T. pallidum* DAL-1 according to the MIGS recommendations [11]

**MIGS ID**	**Property**	**Term**	**Evidence code**^a^
	Current classification	Domain *Bacteria*	TAS [12]
		Phylum *Spirochaetes*	TAS [13]
		Class *Spirochaetes*	TAS [14,15]
		Order *Spirochaetales*	TAS [16,17]
		Family *Spirochaetaceae*	TAS [17,18]
		Genus *Treponema*	TAS [17,19,20]
		Species *Treponema pallidum*	TAS [17,20]
		strain DAL-1	TAS [7]
	Gram stain	negative	TAS [8]
	Cell shape	spiral-shaped	TAS [7]
	Motility	motile	TAS [7]
	Sporulation	none	TAS [8]
	Temperature range	mesophilic	TAS [21]
	Optimum temperature	33–35 °C	TAS [21]
MIGS-22	Oxygen	anaerobic	TAS [22]
	Carbon source	carbohydrates	TAS [23]
	Energy metabolism	chemoorganotroph	TAS [23,24]
MIGS-6	Habitat	host associated	TAS [8]
MIGS-6.3	Salinity	not reported	
MIGS-15	Biotic relationship	parasitic	TAS [8]
MIGS-14	Pathogenicity	pathogenic	TAS [8]
	Host	*Homo sapien*s	TAS [25]
	Host taxa Id	9606	
	Disease	syphilis	TAS [8]
	Cell arrangement	single	TAS [8]
	Biosafety level	2	TAS [26]
	Isolation	amniocentesis	TAS [7]
MIGS-4	Geographic location	Dallas, TX, USA	TAS [7]
MIGS-5	Sample collection time	1991	TAS [7]
MIGS-4.1	Latitude		
MIGS-4.2	Longitude	not reported	
MIGS-4.3	Depth	not reported	
MIGS-4.4	Altitude	not reported	

^a^Evidence codes - IDA: Inferred from Direct Assay (first time in publication); TAS: Traceable Author Statement (a direct report exists in the literature); NAS: Non-traceable Author Statement (not directly observed for the living, isolated sample, but based on a generally accepted property of the species, or anecdotal evidence). These evidence codes are from the Gene Ontology project [27].

*T. pallidum* is a Gram-negative, spiral shaped bacterium 6 to 15 μm in length and 0.2 μm in diameter. *T. pallidum* is an anaerobic non spore-forming motile bacterium that moves by rotating around its longitudinal axis. This movement is powered by endoflagella located in the periplasmic space. The cell wall is composed of a cytoplasmic membrane, a thin peptidoglycan layer, a periplasmic space with endoflagella, and an outer membrane [31].

*T. pallidum* is an obligate human parasite, which does not survive outside its mammalian host and cannot be cultivated continuously under *in vitro* conditions. Optimal conditions for time-limited cultivation in tissue culture consisted of temperature between 33 °C and 35 °C, atmospheric oxygen concentration in the 1.5 to 5% range, 20% fetal bovine serum in the culture medium and the testes extract [21]. Cultivation in tissue cultures resulted in approximately 100-fold multiplication [32,33]. Stable propagation of *T. pallidum* strains can only be achieved in mammalian hosts, usually rabbits.

*T. pallidum* is sensitive to high temperatures [21,34], and is catalase- and oxidase-negative. As a consequence of its small genome, *T. pallidum* has limited metabolic capacity in general [1-3]. Most essential macromolecules are taken up from the host by a number of transport proteins with broad substrate specificity. In total, 113 genes of *T. pallidum* encode proteins involved in transport, which compensate for the absence of genes encoding components of the tricarboxylic acid cycle, oxidative phosphorylation, components for *de novo* synthesis of amino acids, fatty acids, enzyme cofactors and nucleotides [1].

Susceptibility of *T. pallidum* to antimicrobial agents has been tested in tissue culture models followed by testing of treponemal viability using intradermal inoculation of rabbits [35]. No skin lesions were detected following injections of penicillin G: 0.0025 μg/ml; tetracycline: 0.5 μg/ml; erythromycin: 0.005 μg/ml; and spectinomycin: 0.5 μg/ml, indicating that no viable bacteria were present following antibiotic treatment. Unlike penicillin, macrolide regimens have a risk of treatment failure due to chromosomally encoded resistance in *T. pallidum* [36,37].

## Genome sequencing information

### Genome project history

This organism was selected for sequencing on the basis of its increased virulence in rabbits compared to the Nichols strain [1]. The genome project is deposited in the Genomes On Line Database [38] and the complete genome sequence is available at the GenBank (CP003115). The details of the project are summarized in Table 2.

**Table 2:** Project information

**MIGS ID**	**Property**	**Term**
MIGS-31	Finishing quality	finished
MIGS-28	Libraries used	one 454 fragment library, one Illumina
MIGS-29	Sequencing platforms	454 GS20, Illumina GA
MIGS-31.2	Sequencing coverage	45× 454, 91× Illumina
MIGS-30	Assemblers	Newbler 1.0.53.17, Velvet 0.6.05, SeqMan
MIGS-32	Gene calling method	FgenesB, Glimmer, GeneMark, tRNA-Scan, RNAmmer, Rfam
	Genbank ID	CP003115
	Genbank Date of Release	February 8, 2012
	GOLD ID	Gi01869
	Genome Db	BCM-HGSC
	Project relevance	uncultivable human pathogen, medical

### Growth conditions and DNA isolation

Strain DAL-1 was grown in rabbit testis, treponemes were extracted and purified from testicular tissue using Hypaque gradient centrifugation [1,39]. Chromosomal DNA was prepared as described previously [1].

### Genome sequencing and assembly

The genome of strain DAL-1 was sequenced using a combination of Illumina and 454 sequencing platforms (GS20). Pyrosequencing reads (506,607 raw reads of total read length 51,283,327 bp) showing sequence similarity to the Nichols genome sequence [1] were assembled using the Newbler assembler version 1.0.53.17 into 235 contigs (45× genome coverage). Newbler contigs were assembled according to the reference Nichols genome [6] using Lasergene software (DNASTAR, Madison, WI, USA), this assembly reduced the number of contigs to 52 separated by 52 gaps (total length of 19,545 bp). Gaps between contigs were closed using Sanger sequencing. Altogether, 43 individual PCR products were sequenced including 5 XL-PCR products. The PCR products were sequenced using amplification and, when required, internal primers. In addition, 4 libraries of XL-PCR products were prepared and sequenced. The resulting complete genome sequence of strain DAL-1 was considered to be a draft sequence. Additional Illumina sequencing was applied to improve genome sequencing accuracy and the complete DAL-1 genome sequence was compiled from these data. A total of 2,881,557 raw Illumina reads (total length of 103,736,052 bp) were assembled, using the Velvet 0.6.05 assembler [40], into 303 contigs (with 91× average coverage). Out of these 303 contigs, 295 showed sequence similarities to the *T. pallidum* Nichols genome leaving 46,148 bp of *T. pallidum* DAL-1 unsequenced using the Illumina method. Each DAL-1 region not sequenced by Illumina and containing differences from the Nichols genome was resequenced using the Sanger method. In addition, all other discrepancies between the complete DAL-1 genome sequence and the Nichols genome sequence were resolved using Sanger sequencing of both DAL-1 and Nichols strains. Altogether, 15 errors were identified in the 1,093 kb Illumina resequenced region, indicating that the complete DAL-1 genome sequence contained 1 error per 73 kbp. Therefore, the final, corrected, strain DAL-1 genome sequence has an error rate less than 10^-5^.

### Genome annotation

Strain DAL-1 genome was annotated with gene coordinates taken from the Nichols [1], SS14 [2] and Samoa D [4] genomes. These coordinates were adapted and recalculated. Genes identified in the DAL-1 genome were denoted with the prefix TPADAL followed by four numbers to indicate the gene number. Newly predicted genes were identified using the GeneMark and Glimmer programs. In most cases, the original locus tag values of annotated genes were preserved in the DAL-1 orthologs. Newly predicted genes in the DAL-1 genome were named according to the preceding gene with a letter suffix (e.g. TPADAL_0950a).

## Genome properties

**Figure 2:**
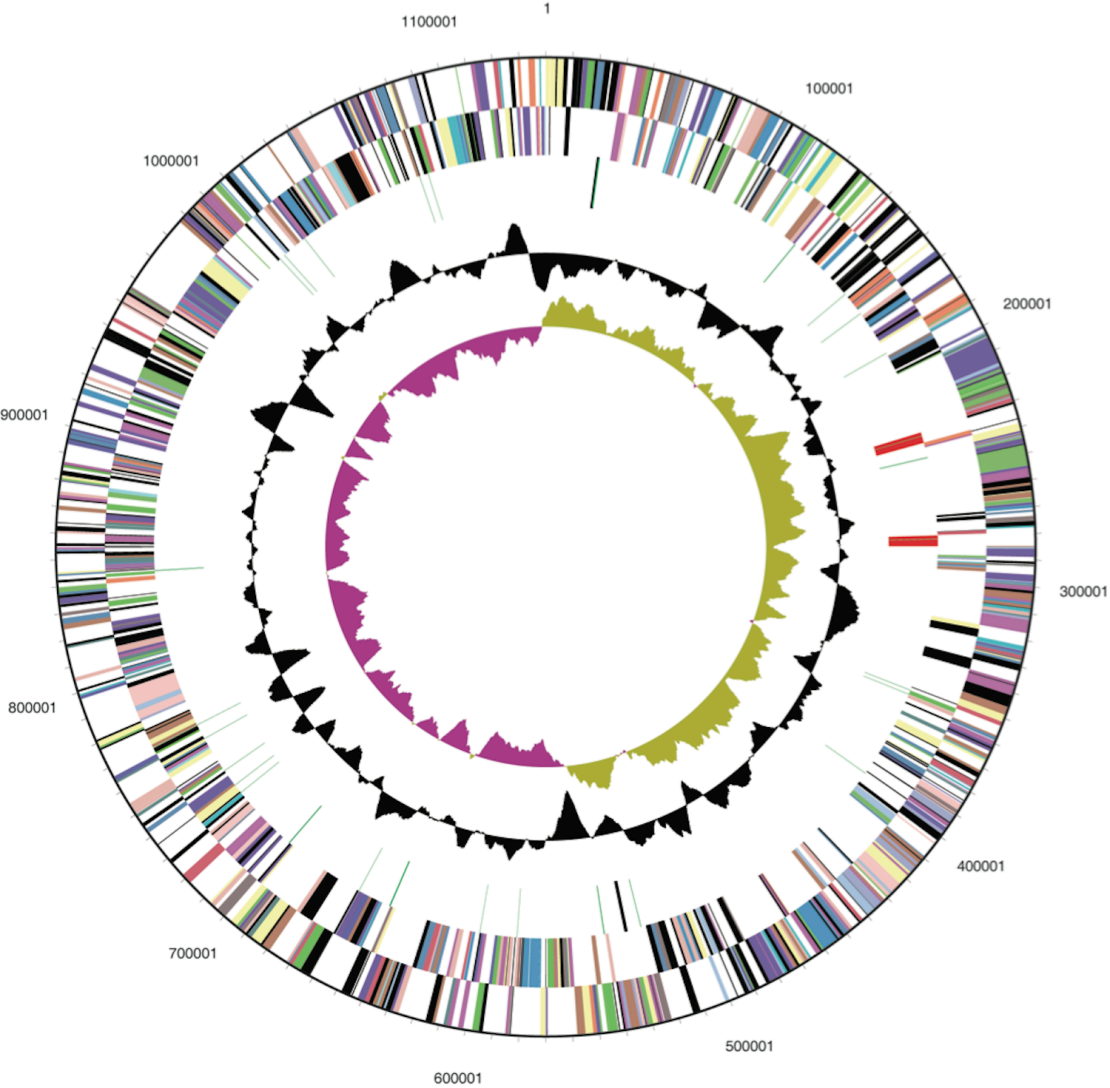
Graphical circular map of the *T. pallidum* strain DAL-1 genome. From the outside to the center: Genes on forward strand (color by COG categories), Genes on reverse strand (color by COG categories), RNA genes (tRNAs green, rRNAs red, other RNAs black), GC content, GC skew. The map was generated with help of DOE Joint Genome Institute [41].

The genome consists of a single circular DNA chromosome, 1,139,971 bp in length. The G+C content is 52.8% (Figure 2, Table 3). Out of the 1,122 predicted genes, 1,068 genes were protein-coding. A set of 54 genes coded for RNA and 9 were identified as pseudogenes. The majority of the protein-coding genes (61.6% of all genes) were assigned a putative function while 33.6% of all genes code for proteins with unknown function. The distribution of genes into COGs functional categories is presented in Figure 2 and Table 4.

**Table 3:** Genome Statistics

**Attribute**	**Value**	**% of Total^a^**
Genome size (bp)	1,139,971	100.00
DNA coding region (bp)	1,073,595	94.2
DNA G+C content (bp)	601,817	52.8
Number of replicons	1	
Extra-chromosomal elements	0	
Total genes	1,122	100.00
RNA genes	54	4.8
rRNA operons	2	
Protein-coding genes	1,068	95.2
Pseudogenes	9	0.8
Protein coding genes with function prediction	691	61.6
Protein coding genes in paralog clusters	178	15.9
Protein coding genes assigned to COGs	756	67.4
Protein coding genes assigned Pfam domains	763	68.0
Protein coding genes with signal peptides	203	18.1
Protein coding genes with transmembrane helices	260	23.2

^a^Based either on the size of the genome in base pairs or the total number of protein coding genes in the annotated genome

**Table 4:** Number of genes associated with general COG functional categories

**Code**	**Value**	**%age**	**Description**
J	117	14.30	Translation, ribosomal structure and biogenesis
K	31	3.79	Transcription
L	59	7.21	Replication, recombination and repair
D	16	1.96	Cell cycle control, cell division, chromosome partitioning
V	7	0.86	Defense mechanisms
T	38	4.64	Signal transduction mechanisms
M	67	8.19	Cell wall/membrane biogenesis
N	50	6.11	Cell motility
U	36	4.40	Intracellular trafficking and secretion
O	47	5.75	Posttranslational modification, protein turnover, chaperones
C	40	4.89	Energy production and conversion
G	44	5.38	Carbohydrate transport and metabolism
E	26	3.18	Amino acid transport and metabolism
F	23	2.81	Nucleotide transport and metabolism
H	23	2.81	Coenzyme transport and metabolism
I	20	2.44	Lipid transport and metabolism
P	26	3.18	Inorganic ion transport and metabolism
Q	3	0.37	Secondary metabolites biosynthesis, transport and catabolism
R	83	10.15	General function prediction only
S	62	7.58	Function unknown
-	366	-	Not in COGs

^a^Several genes were assigned to 2 or more COG categories. In total, 756 protein coding genes were 818-times assigned to COGs.

## Insights into the genome

Sequence changes differentiating the DAL-1 and Nichols genomes were identified mainly in the TPADAL_0136 gene (encoding fibronectin binding protein [42]) and comprised 94 nt changes. In addition, a repeat containing gene, TPADAL_0470 was found to contain 288 nts insertion composed of twelve, 24-bp repetitions. *tpr* genes including *tpr*F (TP0316), *tpr*G (TP0317) and *tpr*K (TP0897) contained 2, 1 and 4 nt changes, respectively. However, the *tpr*K gene was found variable within the DAL-1 strain and therefore the reported 4 nt changes do not refer to the variable *tpr*K region [43]. Tpr proteins are known virulence factors in treponemes [43-48] and the changes in the primary sequence of the protein may be of importance in increased DAL-1 rabbit virulence. In addition to the changes in the above mentioned genes, additional 31 nt changes were found throughout the genome (6 single nucleotide deletions, 3 single nucleotide insertions, 16 single nucleotide substitutions, one 2-nt deletion and one 4-nt deletion). All the indels (with exception of the 4-nt deletion) were found to be located in the G or C homopolymers. Indels resulted in truncation or elongation of several proteins including TPADAL_0012 (hypothetical protein, finally not annotated), TPADAL_0040 (probable methyl-accepting chemotaxis protein), TPADAL_0067 (conserved hypothetical protein), TPADAL_0127a (hypothetical protein), TPADAL_0134a (hypothetical protein), TPADAL_470 (conserved hypothetical protein), TPADAL_0479 (hypothetical protein), and TPADAL_0609 (AsnS, asparagine-tRNA ligase). In addition, TPADAL_0859-860 was identified as a fused protein (TPADAL_0859). Two of the indels in the G or C homopolymers were found in the intergenic regions (IGR TPADAL_0225-226, IGR TPADAL_0316-317). Since G homopolymers, of variable length, affected gene expression rates of *tpr* genes [49], these differences may change the gene expression pattern in the DAL-1 genome. Out of the 16 single nucleotide substitutions, 3 were located in intergenic regions (IGR TPADAL_0126c-0126d, IGR TPADAL_0582-584, IGR TPADAL_0698-700) and three resulted in synonymous mutations (TPADAL_0228, 0742, 0939). The remaining 10 substitutions resulted in 9 nonsynonymous changes in TPADAL_0051 (*prfA*, peptide chain release factor RF1), TPADAL_0065 (probable SAM dependent up methyltransferase), TPADAL_0279 (bifunctional cytidylate kinase/ribosomal protein S1), TPADAL_0433 (*arp*, a repeat containing gene), TPADAL_0674 (encoding conserved hypothetical protein), TPADAL_0720 (*fliY*, bifunctional chemotaxis protein CheC/flagellar motor switch protein FliY), and TPADAL_0854 (encoding conserved hypothetical protein). All of the above listed genes and all the changes in the intergenic regions (potentially affecting gene expression rates) should be considered as potential reason for the observed increased virulence in rabbits.
